# Chemozart: a web-based 3D molecular structure editor and visualizer platform

**DOI:** 10.1186/s13321-015-0101-7

**Published:** 2015-11-19

**Authors:** Mohamad Mohebifar, Fatemehsadat Sajadi

**Affiliations:** Department of Chemistry, Shahid Beheshti University, Tehran, Iran

**Keywords:** Cheminformatics, Web-based, JavaScript, Visualization

## Abstract

**Background:**

Chemozart is a 3D Molecule editor and visualizer built on top of native web components. It offers an easy to access service, user-friendly graphical interface and modular design. It is a client centric web application which communicates with the server via a representational state transfer style web service. Both client-side and server-side application are written in JavaScript. A combination of JavaScript and HTML is used to draw three-dimensional structures of molecules.

**Results:**

With the help of WebGL, three-dimensional visualization tool is provided. Using CSS3 and HTML5, a user-friendly interface is composed. More than 30 packages are used to compose this application which adds enough flexibility to it to be extended. Molecule structures can be drawn on all types of platforms and is compatible with mobile devices. No installation is required in order to use this application and it can be accessed through the internet. This application can be extended on both server-side and client-side by implementing modules in JavaScript. Molecular compounds are drawn on the HTML5 Canvas element using WebGL context.

**Conclusions:**

Chemozart is a chemical platform which is powerful, flexible, and easy to access. It provides an online web-based tool used for chemical visualization along with result oriented optimization for cloud based API (application programming interface). JavaScript libraries which allow creation of web pages containing interactive three-dimensional molecular structures has also been made available. The application has been released under Apache 2 License and is available from the project website https://chemozart.com.

## Background

In the field of computational chemistry, applications which are capable of constructing and viewing 3D structures of molecules play an important role. Such software can be used to by students to understand stereochemical concepts [1]. There are numerous desktop applications available for viewing and building 3D molecules. Avogadro [2], JMol [3], QuteMol [4] and PyMol are few such examples. When it comes to web applications capable of constructing 3D chemical structures, there are not many available.

Today web-based tools are becoming extremely popular. There are numerous benefits which can be derived from them; accessibility, flexible core technologies, platform independency and compatibility are some of them. Most of the web applications which are used for building chemical structures have limited capabilities and most of them are two-dimensional editors such as ChemDoodle [5]. There are several chemical structure editors available powered by Java applets such as JME a free 2D molecule editor java applet [6]; however, they are not compatible with all the browsers. Besides, JAVA needs to be separately installed on the system in order to run these applications. This issue exists for other embedded objects like Flash and Flame [7], a Flash molecular editor is a case in point. There is another web application available to build 3D chemical structures, it is called CH5M3D [8]. It portrays a 3D picture with HTML5 however, it fails to deliver optimum results as it uses canvas 2D context. Because of its limitation, it does not use any shader program. As a result of which three-dimensional rendering cannot be portrayed in the truest sense. Today, it is possible to draw sophisticated graphics that are hardware accelerated by GPU; thanks to HTML5 and WebGL. Furthermore, these components are supported by all modern web browsers, especially mobile browsers.

Applications built using web-based components can be accessed with ease on portable devices such as iPods and smartphones with an additional feature which enable to use it in offline mode too. In today’s tech savvy world, the utilization of chemistry related applications are dramatically growing [9]. This 3D molecule editor targets students at varied levels of study, i.e. high school, college, and graduate school. Along with the students Chemozart targets the chemical professionals, and teachers too with the motive that they can effectively research in any topic and also appropriately solve the queries of their students. We have developed a 3D molecule editor on top of web components which leads to better performance and maintainability. With the help of node.js https://nodejs.org/, it is now possible to use JavaScript outside the web browsers. Both server-side and client-side codes are fully written in JavaScript. By writing both in same language, we seek to enhance integration. Javascript is the language used to develop this software. A Github data visualization factually states that JavaScript is considered to have the most active repositories. As a result, there are diverse packages written in JavaScript with which this software can be bundled.

A chemical toolbox is also needed in order to read different chemical file formats, calculate energy, etc. OpenBabel is considered to be one of the best chemical toolkit which is open-source and it can be easily ported to different languages. There are bindings of OpenBabel in some languages such as Rubabel [10] for Ruby and Pybel [11] for Python. We also made OpenBabel-Node http://mohebifar.github.io/OpenBabel-Node/ to port OpenBabel [12] to node.js. It exposes OpenBabel application programming interfaces (APIs) to many available packages in node.js via a convenient interface. We bundled it with express.js web framework to create a chemical representational state transfer style (REST)ful API. OpenBabel-Node is used to read and write a variety of chemical file formats. Apart from this, it also supports various molecular mechanics force fields and provides optimization of geometry of the molecules.

Chemozart is a web application tool that can be used for viewing and editing of 3D molecular structures. With the help of this web-based platform user can easily create, modify or view the structures of the molecular compounds. With the help of JavaScript and HTML user can easily draw or view the 3D structures of the molecular compounds. The web application represents the molecular structures in both of the client-side and server-side applications. The client-side consists of the UI and the visualization part that helps one to view or edit the structures easily. The convenient user interface offers modifying molecular structures interactively while the visualization part helps in viewing the 3D representations of the molecular compounds.

## Implementation

### Software architecture and interactivity

Chemozart provides a web-based platform for creation, modification and display of molecules. It is available under Apache 2 License which gives power and access to everyone to contribute on extending it. This package consists of a client-side and a server-side application. The client-side application is designed according to Model View ViewModel (MVVM) pattern. It is built with Angular.js which is an MV* client-side framework. It offers service providers, two-way bindings, data models, convenient RESTful resource client service, declarative user interface, etc. The server-side application is designed according to the Model View Controller (MVC) pattern. It is also completely written in JavaScript and uses node.js as the runtime environment. This package is flexible enough to be developed with a well-organized structure. The entire application is written in JavaScript according to ECMAScript 6 which offers classy, flexible and cleaner syntax, a universal way for module definition, etc. Chemozart transpiles ES6 codes to ES5-friendly codes using Babel https://babeljs.io/ for browser support. The stylesheets are originally written in LESS which are compiled to CSS.

The client-side application depends on two major packages which are designed to facilitate the process of binding chemical data to the graphics. Chem.js is a JavaScript package which creates event-based models of molecule, atoms and bonds through an object-oriented approach. It is also used to serialize the molecules in JSON format. Mol3D https://github.com/mohebifar/mol3d is another JavaScript package that displays the structure of molecules and it uses Three.js to render 3D objects with WebGL. It converts the Chem.js objects into Three.js https://github.com/mrdoob/three.js objects according to atoms’ position, atomic radius and CPK coloring convention. The default display mode is “ball and stick” as it is the most convenient model for editing molecules. It is also possible to make arbitrary display modes.

The server-side application depends on OpenBabel-Node which is a submodule of Chemozart, to provide native bindings of OpenBabel in node.js. It is originally written in C++ and makes it possible to use OpenBabel APIs in JavaScript codes. This package is used to build 3D coordinates, calculate the energy of molecules, import and export chemical files including SMILES [13], CML [14], Hyperchem HIN, MDL SD [15], Sybyl Mol2 [16] and PDB [17].

One of the most important aims of Chemozart is to provide a good user experience. So it facilitates building molecules with the help of mouse and keyboard actions or by touch screens devices. Changing the position of atoms is also as simple as dragging them around. All the hidden elements such as periodic table appear with an animation effect that provides a better user experience.

**Fig. 1:**
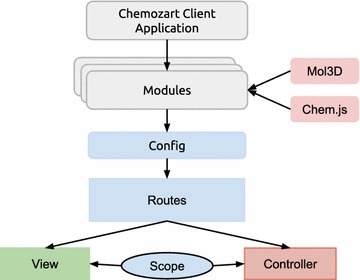
Overview of the client-side application architecture. The client-side application is designed according to MVVM software architecture

While this application focuses on educational purposes, more educational functionalities are planned to be included in future versions. The most important feature is to attach a database to the server-side application to make it possible to share a molecule via a link so that students can share their mind and questions with their teachers. It also facilitates the process of teaching for teachers given that this application works on mobile devices. In addition, more OpenBabel features will be used such as force field clean-up. More complete support for chemical file formats is also planned to be included in future versions (Fig. 1).

### Installation

One of the unique features of this application is the fact that it does not require any installation process and can be accessed online. However, it can be installed locally too. The application requires an existing installation of node.js, npm, bower and grunt. The code is developed and maintained on a Git repository https://github.com/mohebifar/chemozart available on Github. After cloning this repository in any convenient location, dependencies are required to be installed. Note that the client-side dependencies are managed by bower and the server-side dependencies are managed by npm. It is required to run “npm install” and “bower install” commands in a terminal window to install both frontend and backend dependencies. By running “grunt serve” the application will be started and it starts listening on port 9000 by default while a web browser will show up automatically. It is also possible to change the port by changing the OS environment variable “PORT”.

This application requires a web server since it has some functions that use OpenBabel-node. However, it can also be accessed without web server installation but some functionalities that require OpenBabel will not be available such as energy calculation, adding Hydrogens and 3D build, export and import different chemical languages. The different versions of this application are distributed as compressed zip archives and are accessible in the releases menu on the Github page.

## Implementation

### Library development

The client-side consists of the user interface along with a variety of logical modules. It helps to view or edit the structures interactively. The user interface is composed in CSS and HTML, a dynamic stylesheet language called LESS is used in this software. These stylesheets are compiled into CSS which makes it easier to write and maintain stylesheets in big projects. For the views, JADE platform is used that offers inheritance and re-usable functions. Based on MVVM architectural pattern, the molecular models are two-way bound as ViewModel between the controllers and view. It exposes the data objects in such a way that user can view the model structure without being bothered about the back end logic of the model. Working under a framework of Angular.js which is based on MV* architecture, the application firstly reads the view and interprets it as directives and binds the data to a model. The models here are a representation of a molecule drawn or structured by the user working. Further, the models discussed above are originally the atoms and bonds.

The server-side of the application is based on the MVC software architectural pattern that helps to separate this web application into three interconnected parts. This helps in easy viewing of the structure of a molecular compound by separating the inner information from the logics and views. Written in JavaScript, the software uses both node.js and express.js that can be used for fast running of this application and fewer lines of codes to develop.

### Modes

The 3D molecule editor and visualizer works on three modes which are as follows:

- Camera mode
- Editing mode
- Positioning mode

#### Camera mode

In this mode user can rotate any molecular structure just by holding the left button of the mouse and moving it. Its function Pan can also be used just by holding the right button of the mouse and moving it according to requirements. The zoom function of this mode can be accessed when user scroll by holding the middle button of the mouse. This mode works with touch screen devices as well.

#### Editing mode

Editing any molecular structure is quite easy because of the array of options available in this mode. To add atom to any structure user have to click on the empty space while to delete any atom while to delete any atom just right click on the atom itself. By dragging from one atom to another user can add bond. Clicking on a bond adjusts their order. Removing a bond is also possible by right clicking on it.

#### Positioning mode

This mode offers proper positioning of the molecular structure is. In this mode, dragging the atoms on the screen changes the position of them.

### Menus

The menu option of this chemical structure viewing software comprises of three main options which are known as File, Build and Energy.

#### File

The file option of this web application comprises of an array of options which includes New, Open, Save, Delete, Import, Export and Print. User can save or delete a structure easily or even import one from the desktop with the help of these options available in File menu. It supports wide range of file formats to read and write. All the drawn structures are also saved in browser’s local storage.

#### Build

The Build menu comprises of two options which are known as Build 3D and Add Hydrogens. The Build 3D option helps user to generate 3D coordinates for the drawn molecule. Adding Hydrogen functionality to the existing structure is also provided in order to fill out implicit valence spots. The Builder and Hydrogen Adder options use OpenBabel-Node via a RESTful API.

#### Energy

Different molecular mechanics force-field methods like MMFF94, UFF and Ghemical are available to evaluate the energy. By creating the structure and using any of these options the energy shows up on a dialog. Further, this is a common feature of the OpenBabel-Node too.

## Result and discussion

### Technologies and components

**Fig. 2:**
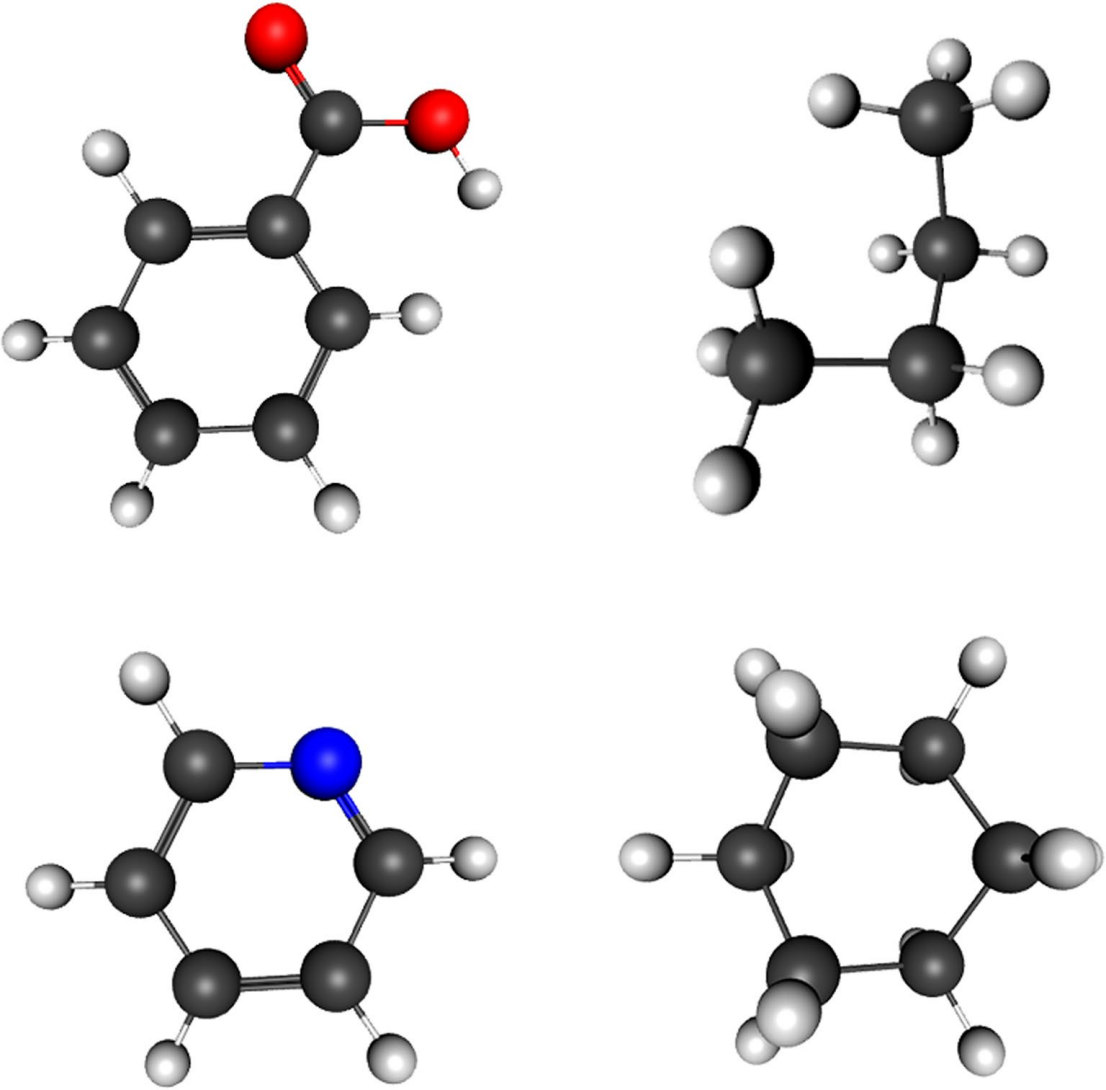
Sample molecules. Chemozart generates high-quality molecular images since it uses WebGL

This web application tool is used for viewing, editing of molecular structures and also view it in the 3D mode. One can easily draw any structure with the help of JavaScript codes and represent it on the HTML5 canvas element with WebGL context. By using standard techniques 3D structure of a molecule can also be viewed. Because of its flexibility in operation and numerous editing and modifying modes, the software is easy to work with and beneficial. With its numerous editing options and easy way to construct or view structures, this chemical software is a good to educate and explaining the detail aspect of 3D geometry in a convenient way. This 3D geometry visualization also helps in performing an in depth analysis of the background of the image and get clear information about the formation of each molecules which is not possible with 2D images with no visual of background images. Analysis of molecular structures and its 3D representations also play an important role in the field of computational chemistry as well. The application does not require any installation and can be accessed online. Interested user can install it locally and continue work without any hassle. It provides a good user experience with the help of keyboard and mouse actions and with the touch screen devices. In accordance with the educational requisites, Chemozart will give the students a clarity about the molecules and their stereochemistry concepts. The biggest advantage of this software is that it assists in educating the students and imparting knowledge to them as it works on portable devices (Fig. 2).

This application is written in JavaScript which is the preferred language for making web applications. Usage of JavaScript enhances the integration as it is used in both client-side and server-side. Moreover, it helps in creating an easy user interface and any one can avail its advanced functionalities just by entering web url in the browser. Now studying a 3D structure of a molecular compound, modifying it or calculating the energy is very easier because of this online chemical software. Chemozart is surely going to be a milestone in the field of constructing 3D images in the field of chemistry to analyze, study and create unique 3D chemical structure images with its user-friendly interface.

### Creating web pages

This application can be accessed in two different environments; development and production. To create a web page development files should be used which make it easier to develop and extend the application. After changing the code files, by running “grunt build” production files will be created in a folder named “dist”. It basically concatenates and compresses the scripts and stylesheets to prevent networks delay caused by transferring all unnecessary characters in the code such as white spaces. To create a chemical editor web page using Chemozart, it is required to determine the angular.js that this web page belongs to a Chemozart application. This can be accomplished by adding the following line to the <body> statement:

<body ng-app = “chemartApp”>

The main element to create a Chemozart web page is the div element with ui-view attribute. It will automatically include the tool bar, status bar, menu and the drawing window to show and edit the molecule. It is accomplished by using angular.js templates.

<div ui-view = “”> </div>

At the end of the body tag, it is required to include all the dependencies in the right order that vary from vendor libraries to each Chemozart modules.

**Fig. 3:**
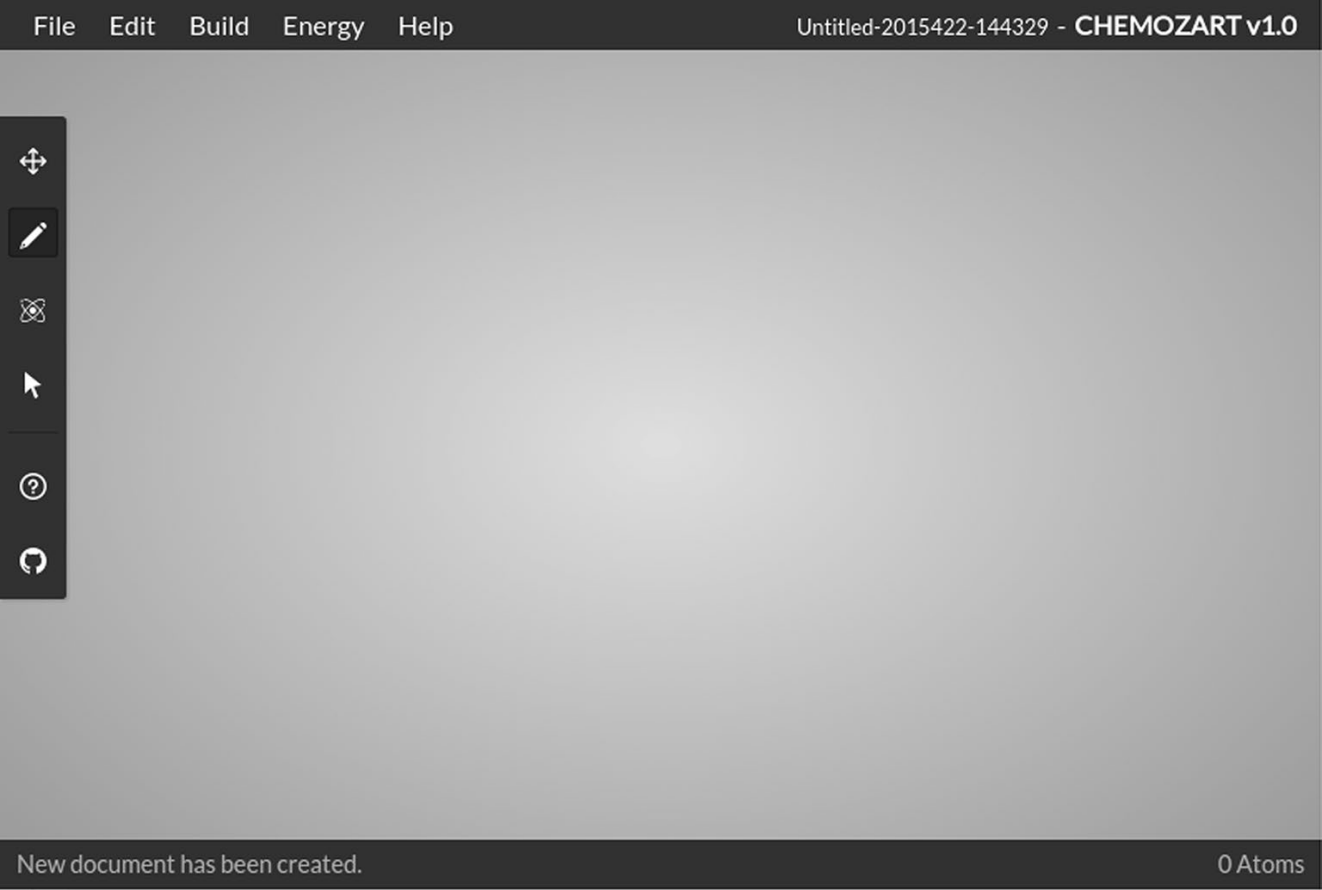
The main page. The main page of Chemozart consists of menu bar, tool bar and a canvas to draw molecules on

Each template can be customized as well as the stylesheets. Templates in JADE and their corresponding stylesheets in LESS are located inside the app or components folder inside the client folder based on their role. Components such as menu bar, tool bar, status bar, periodic table, help window and about window are located in the components folder and the main template that puts these components together is located in the app folder (Fig. 3).

### User-defined functions

This application is built with Angular.js on client-side. All the components and libraries are a directive, a service or a controller. For example, to create a button in the menu bar that adds a carbon on the drawing window the following steps could be used. In the first step, a service must be created.

This service returns a function that creates an instance of “Chem.Atom” that is a class of mol.js module. Then the atomic number should be determined by assigning the value to the “atomicNumber” property. To “position” property determines the atom’s position that should be an instance of “Three Vector”. A singleton of the canvas object can be accessed via a service named “canvas”. To add the created to the drawing window, the atom should be passed to the “addAtom” method on the canvas object. This service should be injected to the main controller (client/app/main/main.controller.js) by adding “addCarbon” to the list of arguments of the main controller function. After injecting the service, it should be assigned to the controller’s scope so it can be accessed in the view.

Finally, a button in the view is needed. To add this button in the menu bar view the JADE file of the menu bar (client/components/menubar/menubar.jade) should be altered as following:

## Conclusions

**Fig. 4:**
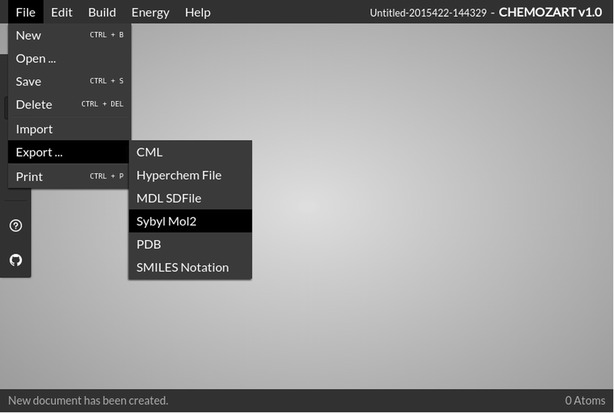
File menu. Chemozart is able to read and write different chemical file formats. It also uses browser’s local storage to store molecular structures

It can be concluded that Chemozart is a chemical web-based application and component which provides the ability to create 3D structure of molecules. The most important feature is that there’s no need to install anything and it can be accessed easily via a URL. Also, it can be beneficial for the educational purposes as well. To catch up with the technology, Chemozart has been designed in a way that it is compatible with the mobile devices. The most up-to-date version of this application is available on the project home page. For the purpose of smooth functioning it is advisable to access Chemozart using the latest versions of browsers across different operating systems (Fig. 4)

## Availability and requirements

Project name: Chemozart

Project home page: https://chemozart.com

Operating system(s): Platform independent

Programming language: JavaScript

Other requirements: An up-to-date web browser

License: Apache2 License

Any restrictions to use by non-academics: None.
